# Round the Hospitals

**Published:** 1919-04-26

**Authors:** 


### ROUND THE HOSPITALS.

NEXT week is crowded with events of nursing interest. Beginning April 28, there will be a Conference at the Mortimer Hall, Mortimer Street, W. 1, in the afternoons and evenings of Monday, Wednesday, and Friday, when nurses will meet in conclave to discuss matters of interest in connection with their profession. Among the subjects to be considered are the College of Nursing with Miss Sparshott as leader of the debate, Poor Law Problems under Miss A. C. Gibson, District Nursing under Queen Victoria's Jubilee Institute, Salaries and Conditions under Lady Bathurst. The meetings are practically free to trained nurses and midwives, but sixpenny stamps must be sent for tickets and catalogue to the Exhibition Secretary, 22 Great Portland Street, W. 1. The Nursing and Midwifery Exhibition will be open at St. Andrew's Hall, Newman Street, Oxford Street, during the whole week from noon to 9 P.M. Sessions relating to midwifery, infant welfare, and kindred topics will be held on Tuesday and Thursday afternoons at St. Andrews Hall.

---

GREAT preparations are being made for the worthy celebration of Nurses' Day on May 1. Many more helpers are needed to ensure its success and many nurses, themselves secure in independence, are offering their services on behalf of their *confrères* less happily situated. None but nurses and those who have intimate knowledge of their case are aware of the extraordinary plights to which members of the profession are frequently reduced in sickness and disablement, partly on account of the fluctuating nature of their income, partly because even when regular the income is apt to be a bare subsistence and leaves no reserves. The entire proceeds of the collections on May 1 are to be set aside for aiding nurses in distress and helping them to make provision for old age. Not only has Queen Alexandra extended her patronage to the Nurses' Flag Day, but Field Marshal Sir Douglas Haig, General Smuts, Sir Alfred Keogh, and Sir A. T. Sloggett have expressed their cordial approval of this appeal to the public.

---

THE Hampstead Stall at the approaching Bazaar at Devonshire House on May 1, 2, and 3, in aid of the Nurses' Fund for Nurses, is likely to be richly furnished, for there are three hospitals in this public-spirited borough, and all are taking a keen interest in it. Besides the Hampstead General Hospital, there are the Mount Vernon Hospital, formerly consecrated to the treatment of soldiers' hearts, but now devoted to the Royal Air Force, and the New End Military Hospital, specialising in trench fever. The matron of this last-named institution is receiving all gifts in kind and money and offers of personal help.

---

SISTERS and nurses from the following hospitals and infirmaries are undertaking stalls at the Bazaar: Chelsea Infirmary, King's College Hospital, the Middlesex Hospital, London Homœopathic Hospital, St. Thomas's Hospital, St. Bartholomew's Hospital, Whipps' Cross Infirmary, Guy's Hospital, Brompton Hospital for Consumption, Queen Mary's Hospital (Stratford), Royal Free Hospital, University College Hospital, Whitechapel Infirmary, and the Hampstead Hospitals. Former members of the staff are earnestly appealed to for gifts of every description, which should be sent to any of the

**ROUND THE HOSPITALS**—(*continued*).

above institutions or to Lady Perry, Chairman of the Nurses' Bazaar Committee, Guy's Hospital, S.E. 1.

The Board of the Leeds General Infirmary have made substantial additions to the salaries of the nursing staff which are much appreciated. Probationers are now to receive £14, £20, £30, and £40 respectively. The new scale of sisters' salaries ranges from £50 to £75 a year, with yearly increment of £5.

A delightful gift which has accrued to the Nation's Fund for Nurses is Seaside Cottage, in Bonchurch, Isle of Wight, which Mrs. Martin Harvey (Miss de Silva) has purchased as a rest-home for members of the College of Nursing. It has ideal surroundings and is in every way well suited to the purpose for which it is intended.

The Nation's Fund for Nurses has received notice of a handsome donation through the will of Lady Webb, the wife of Lieut.-Colonel Sir Henry Webb, Bart., of Llwynarthan, near Cardiff. Lady Webb left £50,000 to her husband, who will devote the whole of it to charities. Among other donations is one of £2,000 to the Nation's Fund for Nurses. It cannot be doubted that this fund will attract many legacies in course of time.

A long list of names was published in the *Times* of the 17th inst. of ladies who have been brought to the notice of the Secretary of State for War for valuable nursing services rendered in connection with the war. They comprise nurses in the Aldershot and the Eastern Commands, the London District, the Northern, the Scottish, the Southern, the Western, and the Irish Commands.

Nothing could more forcibly illustrate the need for registration, including as it does the setting up of a General Nursing Council, than the issue by the Wrexham Orthopædic Hospital of regulations for giving a three-year course of training to probationers with a view to their "filling responsible posts in public hospitals." The iniquity of such an announcement is intensified by the fact that the hospital has been opened under the auspices of the Ministry of Pensions. To project a three-year course of training in a special hospital of this class is, of course, to introduce an element of continual discontent and restlessness leading to abrupt departures, with an ever-increasing difficulty in filling vacancies. Under a properly co-ordinated nursing organisation such an institution, instead of trying to manufacture pseudo-nurses, might fill a useful *rôle* in preparing probationers for regular training who are under the required age, and could secure as staff nurses women anxious to take special training in orthopædic work. We are struck with the strange ignorance of what constitutes a training-school displayed by the medical managers at Wrexham. It is generally assumed that doctors are experts in nursing matters, but this is true, we fear, of a small minority only.

At the Liverpool Royal Infirmary great nursing alterations are in prospect. The question of increasing the off-duty hours of the nursing staff has been long under the consideration of the matron, who, in order to submit a workable scheme to the Committee, has called her sisters into council and asked them to send suggestions that she might utilise with her own. The result of their deliberations has been the repudiation of the eight-hour day principle, which, since it involves three shifts, is considered to be to the disadvantage of patients seriously ill, in whose interests frequent changes ought, if possible, to be avoided. The equivalent of the eight-hour day, a fifty-six-hour week, has, however, been carefully kept in view. Hitherto the sisters' weekly hours on duty have been fifty, staff nurses sixty-three, and probationers sixty-four. Under the proposed re-adjustment it is hoped to bring the working hours down to fifty-six. This will be done by arranging the staff nurses' days off on Sundays, and by an increase of staff sufficient to provide substitutes for probationers' days off in the week. The Committee has shown great consideration and helpfulness in the proposed scheme, part of which will come into operation at once. To secure the extra days off it will be necessary to engage more probationers, and applications from suitable candidates can be at once considered. They will have two days off monthly, sleeping out the previous night should they wish it, and one long evening weekly from 4.30 to 11 P.M., with the usual two hours' daily and extra time on Sunday. The sisters and staff nurses also benefit under this well-worked-out scheme, and all are allowed an 11 P.M. weekly pass if they wish.

Nurses in some of the military hospitals, which, though still kept open, are but scantily provided with patients, have been offered business classes in their spare time, and are making good use of their opportunities. Free tuition in typing, shorthand, French, and other subjects, including, we trust, bookkeeping on the uniform system of accounts, is available in several of these establishments, and the classes are arranged to suit the convenience of nurses coming off duty. The idea is an excellent one, and we should like to see it extended to nurses in isolation hospitals, who in the long slack periods might accomplish great things if they had a teacher.

In face of the satisfactory increase in the salaries of district nurses, the grants now obtainable from County Nursing Associations for school and tuberculosis and health visiting are saving the situation. The minimum commencing salary of a London district nurse is now fixed at £50, and that of a village nurse, partially trained, at £30, in each case with reasonable allowance for board and lodging

**ROUND THE HOSPITALS**—*(continued)*.

and part uniform. It is a great reform, and means in many cases that the nursing of the sick poor will now really be undertaken by the public, instead of, as heretofore, by philanthropic women, devoting their entire time to work which gave them a bare subsistence. Unless, however, some central help had become available for the nursing societies, who are now compelled to raise salaries by a third, they must in many localities have fallen heavily into debt and probably have broken up altogether. The system which has been adopted of giving grants in aid will encourage private liberality and give a great impetus to district nursing.

---

The Funeral Service of Miss Edith Cavell in Westminster Abbey on May 15 will consist of the opening sentences, Psalms, and lesson, with appropriate music, and promises to be one of the most poignant and impressive ceremonies which has ever taken place in that spot, dedicated as it is to so many soul-stirring national memories. The service will begin at noon. Nurses who desire to be present must apply collectively for tickets through the hospital or nursing institute with which they are connected before May 8, to the Hon. Secretary, Funeral Service Executive Committee, 25 Victoria Street, S.W.1. We hope the announcement that "individual applications cannot be entertained unless some special claim to be present can be established" will not exclude nurses who may be unattached to any society.

---

The nursing staff at Paddington Military Hospital have formed a "Recreation Society" in order to provide social evenings once a month. The social part of the gatherings is preceded by the discussion of some matter of professional interest. The first meeting was held on April 2, when Miss Gibson (late matron of Birmingham Infirmary) spoke informally on the College of Nursing. The speaker pointed out the duty of nurses in relation to the traditions of their vocation and to their patients, and dwelt on the advantages the College gives to enterprising nurses of to-day. Refreshments followed the address. Questions were asked and answered.

---

Hope is expressed that members and friends interested in "The Nurses' Union" will embrace the opportunity to attend one or more of the series of Easter re-unions arranged at Miss Dashwood's residence, 18 Palace Garden Mansions, W. A hearty welcome awaits all. The programme of the re-unions is as follows: April 29, 11-12 noon, Miss Dashwood; April 30, 11-12 noon and 3-4, Miss Amy Miller; May 1, Miss Mildred Duff; May 5, 11-12, 3-4, 8-9, Miss Beadon; May 6 and 7, 3-4, Miss Watts (these two meetings will be held at 59 Cromwell Road, S.W., and tea will be provided); May 9, 11-12, 3-4, 8-9, Miss Dawson; May 13 and 14, 8-9 p.m., Mrs. Talbot Hindley.

---

The Conference of the Committee of the Red Cross Societies recently held at Cannes was far more "live" and useful than such meetings generally prove. The British nursing delegates, Miss Gill, R.R.C., Secretary, and Miss Lloyd Still, C.B.E., R.R.C., met in consultation Miss Julia Stimson, Chairman, and Miss Carrie M. Hall, the American nursing delegates, together with the Countess Roussy de Sales (France) and Professor Amelia Anselmi and the Countess Nerina Gigliucci (Italy). One of the main points elucidated was the necessity for an enormous increase in the number of trained workers in all branches of public health work. The connection of this need with the present impulse towards securing shorter hours for nurses is apparent. All the more important nurse training-schools in the country are increasing their accommodation and enlarging the numbers of their probationers, and within a short time the effects of this will be seen in a vastly improved output of trained nurses.

---

The practical result of the Cannes Conference, so far as nursing is concerned, is the decision to found a department of nursing in connection with the International Red Cross Bureau now in course of formation. This department will collect intelligence and undertake propaganda. All National Red Cross organisations are to keep registers of trained nurses available for national or local emergencies, in addition to the registry of voluntary and partly-trained Red Cross workers. The best way of giving additional training to the latter class of workers was discussed, and practical steps in that direction were agreed on. All the delegates were warmly appreciative of the services rendered by voluntary helpers and, though each nation has different ways of utilising their skill and devotion, the desire to retain this body of men and women volunteers for the services of the sick was unanimous.

---

The Cardiff Nursing Association has suffered a great loss recently in the death of its honorary treasurer, Colonel J. A. Jones, who has done much good work for it. In order to link his name in perpetuity with the organisation, a "Colonel Jones Memorial Fund" has been started, and already over £700 has been subscribed to it.

---

The Mayor of Exeter, Sir James Owen, presiding at the ninth annual meeting of the Exeter District Nursing Association, commented on the fact that the support accorded to it was comparatively small. The number of cases dealt with was in excess of all previous years, and a maternity home had been secured in which twenty-six cases had been nursed. It is much to be regretted that the parish authorities should have refused to affiliate with the association, so that all the parishes in the city area could have been consolidated for sick-nursing purposes. The Mayoress expressed the bitter disappointment of her depôt at the refusal of the City Council to allow a Flag Day to be held on behalf of the association.

